# Structural Patterns of Antibiotic Shortages: A Cross-National Analysis of Systemic Antibacterials

**DOI:** 10.3390/antibiotics15060571

**Published:** 2026-06-03

**Authors:** Oana-Teodora Chirac, Adriana-Elena Tăerel, Mihaela Dinu, Robert Ancuceanu

**Affiliations:** 1 Department of Pharmaceutical Botany and Cell Biology, Faculty of Pharmacy, “Carol Davila” University of Medicine and Pharmacy, 050474 Bucharest, Romania; oana-teodora.chirac@drd.umfcd.ro (O.-T.C.); mihaela.dinu@umfcd.ro (M.D.); robert.ancuceanu@umfcd.ro (R.A.); 2 Department of Management and Pharmaceutical Marketing, Faculty of Pharmacy, “Carol Davila” University of Medicine and Pharmacy, 020956 Bucharest, Romania

**Keywords:** antibiotic shortages, multinational shortage recurrence, cross-national analysis, EMA critical medicines

## Abstract

**Background/Objectives**: Drug supply disruptions represent an increasingly serious problem for health systems worldwide, with systemic antibiotics among the most frequently affected therapeutic categories. Although regulatory authorities have repeatedly signaled this risk, comparative studies analyzing patterns of antibiotic shortages across multiple countries simultaneously remain scarce. **Methods**: We performed a cross-sectional comparative analysis based on data from public national shortage registries in seven jurisdictions: Belgium, France, Germany, Romania, Spain, the United States (FDA), and the Kingdom of Saudi Arabia. All records corresponding to systemic antibiotics in ATC group J01 were extracted, harmonized, and analyzed, with the active substance (INN) as the unit of analysis. The association between critical drug status according to the EMA list and the multinational recurrence of shortages was assessed using chi-square tests, the Mann–Whitney U test, and multivariate logistic regression. To verify the robustness of the results, a sensitivity analysis was also performed using alternative thresholds for jurisdictions. **Results**: A total of 350 shortage records were mapped, corresponding to 64 unique active pharmaceutical ingredients. On average, each active substance was reported as out of stock in 3.48 jurisdictions (SD = 1.46). Macrolides (J01F) and quinolones (J01M) exhibited the widest geographic spread of shortages. Antibiotics included on the EMA’s list of critical medicines were reported as missing in multiple countries simultaneously significantly more frequently than those not included on this list (82.86% vs. 37.14%; χ^2^ = 71.99, *p* < 0.001; Cramer’s V = 0.454). In the multivariate logistic regression model, EMA critical medicine status remained an independent predictor of multinational recurrence of shortages (OR = 8.29; 95% CI: 4.93–13.94; *p* < 0.001), while the injectable route of administration did not reach the threshold for statistical significance (OR = 0.78; *p* = 0.341). Sensitivity analysis confirmed that this association remains statistically significant regardless of the threshold chosen. **Conclusions**: Shortages of systemic antibiotics tend to occur simultaneously in multiple countries, and drugs designated as critical by the EMA are disproportionately affected. The results suggest that the identified weaknesses are not specific to a single health system but reflect structural fragilities in international antibiotic supply chains. This underscores the need for internationally coordinated strategies, both for monitoring the availability of essential antibiotics and for preventing and managing shortages.

## 1. Introduction

Interruptions in the supply of medicines are a significant challenge with intricate consequences for global public health systems [1]. In recent decades, they have become increasingly frequent and often difficult to predict. Despite the measures taken so far, they continue to impact national healthcare systems, patients, healthcare professionals, regulatory authorities, and all actors involved in the supply chain, including manufacturers, distributors, pharmacies, and hospitals. This situation places significant pressure on medical decisions and access to treatment [2,3]. Limited access to treatment has serious implications for both public health systems and individual patients. Thus, drug shortages can cause delays in initiating treatment and the use of less effective or less safe therapeutic alternatives (e.g., second-line treatment options). Such delays or therapeutic switches can lead to increased therapy costs, and, in the case of systemic antibiotics, an increased risk of microbial resistance [4,5].

In this context, we conducted a structural analysis of antibiotic (ATC code J01) shortages in a number of seven states from three continents. Antibiotics are a key group of medicines [6] that are essential for public health systems [7]. They are the primary therapeutic option in the treatment of bacterial infections, being indispensable in the management of severe infections, especially in the hospital environment [8,9]. Their continuous availability is, therefore, absolutely necessary for the optimal functioning of healthcare systems. In recent years, regulatory authorities, including the European Medicines Agency (EMA), have repeatedly highlighted the risk of supply disruptions for certain antibiotics, particularly during times of increased seasonal respiratory infections [10].

The existing vulnerabilities in global pharmaceutical supply chains have been made more visible in the context of the COVID-19 pandemic [11]. When in the early phases of the pandemic, the empirical use of antibiotics in the management of patients with severe respiratory infections led to an increase in demand for certain antibiotics. This phenomenon contributed to the emergence or worsening of supply disruptions [12]. On the other hand, logistical restrictions and the disruption of normal production chain operations induced by health authorities during that period have exacerbated the risks associated with the availability of essential medicines [13], including a number of antibiotics.

Recognizing the importance of these medications for the health systems of the European Union (EU) member states, EMA has published a list of drugs considered critical at the European Union level; this list includes multiple antibiotics frequently used in clinical practice [14]. Such identification of critical medicines is useful in monitoring and prevention of supply disruptions for products of major importance to public health [15,16], including antibiotics.

In this context, a comparative analysis of the available data from national shortage registers can provide valuable information regarding the patterns of supply disruptions and can contribute to identifying common vulnerabilities in supply chains. Although numerous studies have analyzed drug shortages at the national level, there are relatively few comparative analyses that integrate data from multiple territories to assess the transnational recurrence of antibiotic shortages and to open avenues for analyses of national regulatory legislative contexts alongside market access policies from the perspective of union policies that can help prevent shortages, especially for drugs declared essential [17].

**Figure 1:**
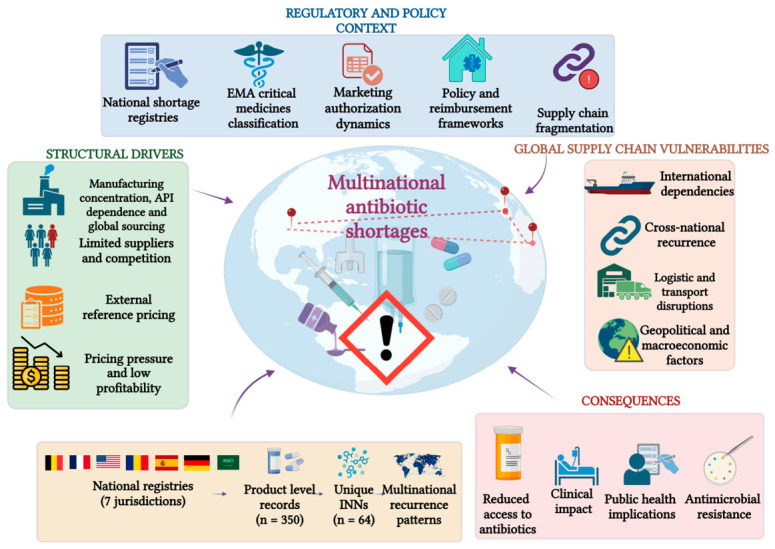
Conceptual framework illustrating the structural determinants associated with multinational antibiotic shortages. The figure summarizes the interaction between regulatory and policy factors, pharmaceutical market dynamics, global supply chain vulnerabilities, and structural drivers contributing to the recurrent reporting of antibiotic shortages across multiple jurisdictions.

The selection of data regarding disruptions in the supply of systemic antibiotics for several European countries, as well as the United States of America, has allowed for a comparative analysis in a context characterized by interconnected regulatory systems and global supply chains. The member states of the European Union are integrated into a common regulatory framework for medicines, coordinated by EMA; however, the mechanisms for reporting and managing shortages, as well as pricing policies, remain largely the responsibility of national authorities [18,19]. Therefore, national registers may reflect both local peculiarities of the pharmaceutical market and common vulnerabilities of supply chains. With the inclusion of the FDA shortage database for systemic antibiotics, we intended to broaden the framework for observing patterns beyond Europe. This allows for a comparison with one of the largest pharmaceutical markets in the world, and such a comparative analysis helps identify common patterns in supply disruptions. These patterns are more likely to reflect structural problems in the worldwide manufacturing and distribution of antibiotics, rather than issues specific to a single country [20]. Therefore, in this study we focused on systemic antibiotics (ATC J01), using data from multiple national shortage registers. In the present study, the term “structural” refers to recurrent and systemic shortage patterns associated with persistent characteristics of pharmaceutical markets, supply chains, regulatory systems, and manufacturing dependencies, rather than isolated or short-term logistical disruptions. A conceptual overview of the structural determinants contributing to multinational antibiotic shortages is presented in Figure 1.

The study aimed to evaluate the distribution of antibiotics reported in shortage across different jurisdictions; investigate the association between the EMA critical medicine status and the multinational recurrence of shortages; and identify the factors associated with shortages in multiple countries. Our results show that shortages happen multiple times in different therapeutic classes and in different places. Logistic regression made it possible to explore factors that were independently linked to shortages reported at the multinational level.

## 2. Results

### 2.1. The Distribution of Antibiotic Shortages Across Countries and Therapeutic Classes

Our data set included a total of 350 reported shortage records for antibiotics belonging to therapeutic class J01; these were identified based on official shortage reports in the seven jurisdictions included in this analysis (Belgium, France, Germany, Romania, Spain, the U.S./FDA, and Saudi Arabia). The unit of analysis was the active substance (INN), with each antibiotic included only once in the final dataset. The number of countries in which shortage was reported for each INN ranged from 1 to 6, with a mean of 3.48 (SD = 1.46). This indicates a relatively broad distribution of antibiotic shortages in the analyzed jurisdictions. Most antibiotics were reported as unavailable in at least three countries. The high proportion of antibiotics reported in at least three jurisdictions suggests a substantial level of multiple-country recurrence, supporting the presence of structural (rather than merely local) shortage patterns.

**Figure 2:**
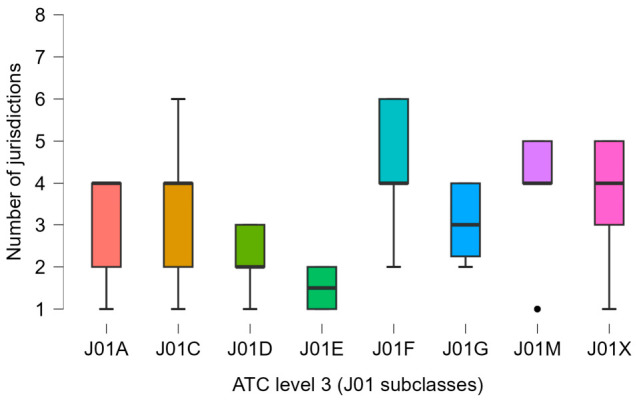
Distribution of the number of jurisdictions reporting shortages according to ATC level 3 subclasses of systemic antibiotics (J01). Analyses were conducted at the INN level using the harmonized dataset. The figure illustrates the variability in the geographic recurrence of shortage reports across therapeutic subclasses, with higher values indicating antibiotics reported in a larger number of jurisdictions.

To evaluate whether antibiotic shortages are structurally concentrated at the therapeutic level, we analyzed their distribution across ATC level 3 classes. This revealed substantial heterogeneity among the examined classes, as presented in Figure 2. The highest average values for the number of jurisdictions affected by shortages were recorded for macrolides (J01F), with an average of 4.64 jurisdictions, followed by quinolones (J01M), with an average of 4.00 jurisdictions, and other antibacterials (J01X), with an average of 3.95 jurisdictions. At the opposite pole, sulfonamides (J01E) had the lowest geographical extent of shortage, with an average of 1.50 jurisdictions, followed by cephalosporins (J01D), with an average of 2.20 jurisdictions. The clustering of shortages in certain ATC categories, such as macrolides and quinolones, indicates that structural weaknesses might be associated with common production or demand traits within these therapeutic groups.

### 2.2. Association Between Shortages and EMA Critical Medicines

Including the European Medicines Agency’s (EMA) list of critical medicines in the analysis revealed a significant association between a medicine’s critical status and the likelihood that an antibiotic would be reported as in short supply across multiple jurisdictions. Antibiotics included on the EMA list showed a more pronounced tendency to be affected by recurrent shortage internationally compared to those not classified as critical. According to the contingency table, 82.86% of the antibiotics classified as critical by the EMA were reported as being in short supply in at least three jurisdictions, compared to 37.14% of the antibiotics not included on the critical medicines list. In absolute terms, 203 of 245 antibiotics classified as critical exhibited multinational shortages, compared to 39 of 105 non-critical antibiotics.

The chi-square test, according to the values presented in Table 1, indicated a statistically significant association between EMA critical drug status and the distribution of shortage across multiple countries (χ^2^ = 71.99; df = 1; *p* = 2.16 × 10^−17^). The effect size, estimated by Cramer’s V coefficient, was 0.454, indicating a moderate to large effect according to conventional interpretation thresholds. This result suggests that critical drug status represents a relevant structural factor associated with the geographic extent of shortages for antibiotics in the J01 class.

Overall, the results support the hypothesis that antibiotics considered essential for public health are more vulnerable to recurring international supply disruptions, possibly reflecting increased pressure on supply chains for molecules of major therapeutic importance.

### 2.3. Differences in the Territorial Extent of Shortages Between EMA-Designated Critical and Non-Critical Antibiotics

A comparison of the number of jurisdictions in which supply disruptions were reported revealed that antibiotics classified as critical by the EMA are affected differently from those not classified as critical, with the difference being statistically significant. Since the number of countries in which a shortage manifested did not follow a normal distribution, differences between groups were assessed using the nonparametric Mann–Whitney U test. The results indicated that antibiotics included on the EMA’s list of critical medicines were reported as being in supply disruption in a significantly higher number of territories compared to non-critical antibiotics (Mann–Whitney U = 6900, *p* < 0.001). The mean number of affected countries was 3.86 (SD = 1.29) for critical antibiotics, compared with 2.59 (SD = 1.45) for non-critical ones. The effect size, estimated using the rank-order correlation (r = 0.464), indicates a moderate to large effect, suggesting that the observed difference is relevant not only statistically but also clinically, as these results raise a red flag regarding the fact that antibiotics considered essential for public health tend to exhibit shortages with a broader geographic distribution, simultaneously affecting a larger number of jurisdictions.

**Figure 3:**
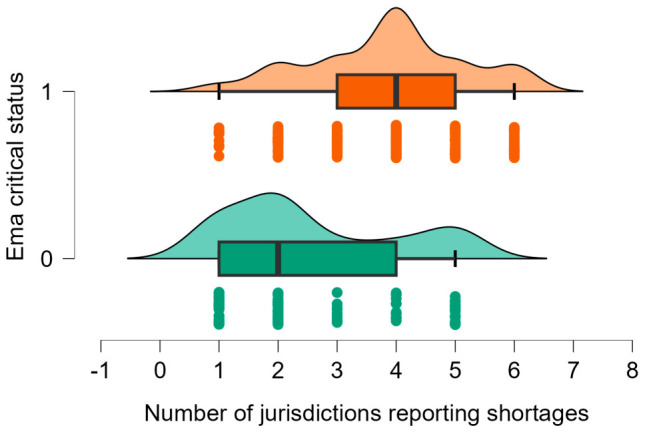
Raincloud plot showing the distribution of the number of jurisdictions reporting shortages according to EMA critical medicine status. Orange denotes antibiotics classified as critical by the European Medicines Agency (EMA), whereas green denotes non-critical antibiotics. Analyses were conducted at the INN level. Individual points correspond to unique antibiotic active substances reported across jurisdictions.

The raincloud plot shown in Figure 3 shows a shift toward higher values in the number of jurisdictions for antibiotics classified as critical by the EMA, indicating a wider territorial expansion of shortages compared to non-critical antibiotics, which more frequently present values focused in the lower range (1–3 jurisdictions). Altogether, the results support the hypothesis that the EMA’s designation of a drug as critical is associated with a broader geographical extension of shortages, which may reflect the occurrence of structural weak points in the supply chains for antibiotics of major therapeutic value.

### 2.4. Country-Level Comparison of EMA Critical Shortages

The allocation of antibiotics categorized as critical-importance medicines by the European Medicines Agency (EMA) was relatively evenly spread across the jurisdictions analyzed. The ratio of critical antibiotics reported as being in shortage fluctuated within a relatively narrow range, from 67.13% in Saudi Arabia to 78.26% in the US/FDA, with the other jurisdictions showing equivalent values: Belgium 70.45%, France 68.18%, Germany 68.29%, Romania 77.78%, and Spain 68.75%. The chi-square test presented in Table 2 did not indicate statistically significant differences between jurisdictions regarding the proportion of critical antibiotics reported as shortage (χ^2^ = 2.723; df = 6; *p* = 0.843). The effect size, estimated using Cramer’s V coefficient, was 0.088, indicating a very small effect according to conventional interpretation thresholds.

The results obtained and presented in Table 2 reveal that the shortages affecting antibiotics deemed critical by the EMA tend to manifest in a relatively similar manner across the jurisdictions analyzed, without being primarily explained by country-specific factors. This may signify that the availability of these essential antibiotics is influenced by structural factors common at the international level.

### 2.5. Multivariable Analysis

This analysis sought to assess whether the European Medicines Agency’s (EMA) designation of a drug as “critical” is independently associated with the geographic spread of shortages; a logistic regression model was constructed with the multinational recurrence of shortages (binary variable, defined as the reporting of shortages in at least three jurisdictions) as the dependent variable. The model included the EMA critical medicine status and route of administration (oral versus injectable), given the clinical relevance of access to parenteral antibiotics in the treatment of severe infections. The adjusted model demonstrated a significant improvement in fit compared to the null model (Δχ^2^ = 70.44, *p* < 0.001), indicating an adequate ability to explain the variability of the phenomenon. The model’s explanatory power was moderate (Nagelkerke R^2^ = 0.257). The data are presented in Table 3.

Critical status according to the EMA was independently associated with a significantly higher probability of shortage with multinational distribution. Antibiotics included on the EMA list were approximately eight times more likely to be reported as shortage in at least three jurisdictions compared with non-critical antibiotics (OR = 8.29; 95% CI: 4.93–13.94; *p* < 0.001). On the other hand, the injectable route of administration was not significantly associated with the probability of multinational recurrence of shortage after adjusting for EMA status (OR = 0.78; 95% CI: 0.47–1.30; *p* = 0.341), and these results are presented in Table 4. No multicollinearity was observed among the predictors included in the model, with variance inflation factor (VIF) values very close to 1 for both: EMA critical status (VIF = 1.007) and the injectable route (binary variable; VIF = 1.007), indicating statistical independence among the explanatory variables. At the same time, the model’s predictive performance was considered appropriate, with an overall accuracy of 76.9% and good discriminatory power (AUC = 0.769). From a clinical standpoint, the results suggest that antibiotics that are considered essential for the therapy of severe infections present a high susceptibility to recurring shortages worldwide, regardless of the route of administration. This pattern may mirror the increased strain on global supply chains for therapeutic molecules of critical relevance.

### 2.6. Sensitivity Analysis: The Effect of Variation in the Threshold on the Main Results

To assess the robustness of the results relative to the threshold chosen for defining multinational shortages, a sensitivity analysis was conducted using alternative values for the threshold defining the dependent binary variable, which examines the number of countries in which a specific INN in the ATC category J01 is reported as a shortage in ≥2 and, respectively, ≥4 jurisdictions. The association between critical drug status according to the EMA list and the multinational recurrence of shortages remained statistically significant at all three tested thresholds (≥2 jurisdictions: χ^2^ = 7.81, *p* = 0.005, Cramer’s V = 0.349; ≥3 jurisdictions: χ^2^ = 8.27, *p* = 0.004, Cramer’s V = 0.359; ≥4 jurisdictions: χ^2^ = 4.47, *p* = 0.034, Cramer’s V = 0.264). The proportion of antibiotics classified as essential by the EMA and falling into the multinational shortage category consistently exceeded the proportion of non-critical antibiotics, regardless of the threshold applied (81.8% vs. 45.2% for ≥2; 57.6% vs. 19.4% for ≥3; 39.4% vs. 12.9% at ≥4). The threshold of ≥3 jurisdictions generated the largest effect size (Cramer’s V = 0.359) and the highest odds ratio for EMA critical status in the logistic regression model (OR = 3.75). On the other hand, the injectable route of administration remained statistically insignificant as an independent predictor of multinational recurrence at the original threshold (OR = 0.98), a result consistent with the primary analysis reported in this study. These results demonstrate that the primary analysis is reliable and suggest that the correlation identified is not an artificial construct.

### 2.7. Policy Implications

The findings of this study emphasize the necessity to develop coordinated policies to strengthen the resilience of supply chains for essential antibiotics [21]. The observed association between a drug’s designation as “critical” by the EMA and the multinational recurrence of supply disruptions suggests that certain molecules of major clinical importance may be exposed to structural vulnerabilities in the pharmaceutical market [22], which would strongly justify the implementation of preventive measures at both the national and international levels. One way to help is to encourage the production of generic antibiotics through economic means that make sure these products can be sold for a long time. Updating pricing policies or the tax system for essential medicines could encourage the production of generic antibiotics through economic measures that ensure their long-term availability [6,23]. Additionally, maintaining minimal strategic stockpiles based on consumption for critical antibiotics could help mitigate the impact of temporary disruptions on clinical practice, especially for treatments used in severe infections or acute cases. Increasing transparency in supply chains for active pharmaceutical ingredients is also crucial, including identifying dependencies on a limited number of manufacturers [24] or the concentration of active ingredient suppliers in limited geographic regions that may face (wholly or partially) unfavorable political contexts [25]. While international harmonization and efforts to facilitate collaboration between jurisdictions in drug manufacturing [26] might sometimes disadvantage local markets, the overall findings support the effectiveness of integrated approaches. Such approaches could combine pharmaceutical policy tools, monitoring mechanisms, and international cooperation to reduce the risk of supply disruptions for antibiotics that are essential in medical practice [27].

## 3. Discussion

The results of this study highlight the existence of particular patterns in terms of antibiotic supply disruptions reported in the national registries analyzed. Although most antibiotics in the ATC group J01 were reported to be in shortage in a distinct manner across the jurisdictions included in the analysis, suggesting predominantly local events, the analysis also identified a small group of molecules with multinational recurrence. This result suggests that, in some cases, antibiotic shortages may reflect vulnerabilities in global antibiotic production and distribution chains rather than isolated country-specific issues. Among the antibiotics with the widest geographical spread of shortages were amoxicillin and azithromycin, reported in six out of seven jurisdictions, followed by levofloxacin and vancomycin, reported in five out of seven jurisdictions, bringing to light the particular clinical significance of these molecules [28].

Supply disruptions for first-line antibiotics such as amoxicillin can significantly influence prescribing practices. A recent study conducted in Italy demonstrated that the unavailability of amoxicillin resulted in a significant increase in the prescription of broader-spectrum antibiotics, including amoxicillin–clavulanate and third-generation cephalosporins. This shift partially undermines the progress of antimicrobial stewardship programs [29], which have been a focus of recent efforts involving public education, national legislative changes, and the establishment of legal frameworks to restrict uncontrolled access to systemic antimicrobials [30,31]. Similarly, azithromycin is a macrolide widely used in the treatment of community-acquired respiratory and other common bacterial infections and is frequently recommended in clinical practice guidelines [32,33]. This contributes to sustained global demand for azithromycin. During the COVID-19 pandemic, its off-label use in viral respiratory infections increased substantially, particularly during the early stages of the pandemic when its therapeutic role remained uncertain [34]. This temporary surge in demand likely placed additional pressure on already vulnerable supply chains, especially given the concentration of generic antibiotic manufacturing and API production in a limited number of geographic regions [35].

The identification of vancomycin among the antibiotics reported in shortage across multiple jurisdictions is clinically important given its central role in the treatment of severe bacterial infections [36]. Disruptions in vancomycin supply may require the use of more expensive or less effective therapeutic alternatives, potentially affecting the management of hospitalized patients with severe infections. These findings further emphasize the importance of maintaining resilient supply chains and continuously monitoring the availability of essential antibiotics, particularly for critical hospital-based therapies [32,37,38].

Recently published papers show that disruptions in the supply of antimicrobials are a persistent global problem, with antibiotics being among the classes of drugs most frequently affected by shortages. The causes of these disruptions are complex and include manufacturing problems, economic pressures on generic drug manufacturers, and the relocation of active pharmaceutical ingredient production to a limited number of geographic regions [13,39]. In addition, fluctuations in demand and limited production capacity can amplify the vulnerability of antimicrobial supply chains [40]. Antibiotic shortages can significantly impact clinical outcomes of patients with bacterial infections, as they can require the use of alternative therapies, can cause increased treatment costs, and potentially contribute to the development of antimicrobial resistance [41,42].

Although important international efforts are focused on limiting antimicrobial resistance, the growing unpredictability of antibiotic shortages [36] may indirectly weaken these strategies. In the absence of coordinated international mechanisms capable of ensuring the stable availability of essential antimicrobials, recurrent supply disruptions may compromise both antimicrobial stewardship efforts and the long-term resilience of healthcare systems [43,44].

Including the EMA list of critical medicines [15] in our analysis revealed a significant association between such an inclusion (the critical medicine status) and the likelihood of multinational shortages. The supply of antibiotics included in the EMA list was more often disrupted in multiple countries than antibiotics not included in the EMA list. In the multivariable logistic regression model, EMA critical status remained an independent predictor of multinational shortage occurrence; antibiotics on the EMA list had approximately eight-fold higher odds of being reported in shortage in at least three jurisdictions than other antibiotics. This is particularly worrying, since the absence of such critical status antibiotics can have significant clinical consequences [45].

Although shortages of injectable antibiotics are often emphasized in the literature due to their importance in hospital care [20]. The parenteral route of administration was not associated with multinational shortage occurrence after adjustment for EMA critical status. Nevertheless, in the recent literature, it has been shown that injectable antibiotics are often affected by supply chain disruptions, especially when manufacturing is limited to a small number of companies [22].

We did not find significant differences in the distribution of critical antibiotics reported to be affected by shortages in our comparative analysis. This suggests that supply disruptions for essential antibiotics are relatively consistent across different regulatory systems and pharmaceutical markets. Thus, the observed shortages do not seem to be determined exclusively by country-specific factors, but rather reflect structural vulnerabilities in international supply chains. This holds true particularly at the EU level, where, although each country has its own market access legislation, most member states use external reference pricing systems. This can contribute to the emergence of shortages through the very low pricing policy for generic antibiotics, which disincentivizes manufacturers and may lead to product withdrawal from the market or to a limitation of production capacity [46].

The therapeutic classes most frequently involved in cross-border shortage were macrolides and penicillins, characterized by both a high number of affected molecules and a high frequency of reports across the states included in our study [47].

Taken together, these observations further highlight the role of economic and regulatory pressures in shaping the long-term sustainability of essential antibiotics. In particular, persistent downward pricing pressures and heterogeneous reimbursement policies may reduce the commercial viability of older generic antibiotics and indirectly contribute to recurrent supply instability [38,48,49,50,51]. This result is consistent with observations from other studies on antibiotic shortages [47,52], which have shown that certain therapeutic classes may be more vulnerable to supply chain disruptions due to the particularities of production processes or demand dynamics [22]. The macrolide drug class was involved in consumption fluctuations during the COVID-19 pandemic, when, in particular, azithromycin was frequently administered empirically to patients with viral pneumonia, due to its potential anti-inflammatory effect and the hypothesis of preventing bacterial co-infections [53], and this could partly explain the multinational vulnerability of the supply chain.

Logistic regression analysis highlights that the status of a critical medicine according to the EMA list is an important factor associated with the early monitoring of molecules that, due to their use and importance, carry an intrinsic risk of shortages. The results show that antibiotics included on the list of critical medicines are associated with a much higher risk of being reported as shortage in multiple jurisdictions, which also implies a global threat. This association may be a consequence of the fact that antibiotics considered essential are frequently used in clinical practice [9,32].

The results of this study have significant implications for health policies. The identification of essential antibiotic shortages across multiple countries suggests the need for internationally coordinated strategies to monitor and manage supply chain risks. Recent initiatives by regulatory authorities, such as developing lists of critical medicines and strengthening shortage reporting mechanisms, represent important steps in this direction [15,16].

## 4. Materials and Methods

### 4.1. Study Design and Data Sources

A cross-sectional comparative analysis was performed to evaluate reported antibiotic shortages identified in national shortage registries. The study focused on systemic antibiotics belonging to the ATC J01 group (systemic antibacterials) according to the Anatomical Therapeutic Chemical (ATC) classification system.

In this study, the terms “shortage” and “supply disruption” are considered equivalent and are used synonymously to describe reported interruptions in the availability of antibiotics in national registers.

In this context, structural patterns refer to recurrent shortage characteristics observed across multiple jurisdictions, beyond isolated local regulatory influences. These patterns were explored through three complementary analytical dimensions: (1) the distribution of shortages across ATC level 3 therapeutic subclasses, (2) the recurrence of shortages across countries at the INN level, and (3) the identification of predictors associated with multinational shortage recurrence.

The analysis was performed entirely on the basis of publicly available data from official registers of shortages and was intended to identify common patterns of the phenomenon, rather than investigate the specific causal mechanisms of each market. By comparatively integrating data from multiple jurisdictions, the study assessed the extent to which certain therapeutic classes or categories of antibiotics have similar shortage profiles, suggesting the existence of structural characteristics of the shortage phenomenon for antibiotics in the ATC group J01. The selection of the ATC J01 therapeutic group (systemic antibacterials) for this analysis was made in view of the major clinical importance of antibiotics and the frequency of reports of supply disruptions for this category of medicines in recent years. Regulatory authorities, including EMA [10,54], have repeatedly issued communications and alerts regarding the availability of antibiotic stocks, particularly during periods of increased incidence of seasonal respiratory infections. These communications pointed out the risk of stock shortages for essential antibiotics used in current clinical practice.

An additional relevant context is the COVID-19 pandemic, when, as mentioned above, in the early stages of the pandemic, the empirical use of antibiotics in the management of patients with severe respiratory infections led to increased demand for certain antibiotics and, in some cases, to disruptions in supply chains. Such circumstances revealed existing vulnerabilities in the global production and distribution of antibiotics.

Antibiotics are included in regional or national lists of essential medicines, such as the EMA’s Critical Medicines List [15] or the World Health Organization’s (WHO) List of Essential Medicines [55], a fact that reflects their central role in the therapeutic management of bacterial infections and thus in the operation of the healthcare systems. In critical clinical situations, the rapid availability of suitable antibiotics is crucial to prevent severe complications and reduce mortality associated with bacterial infections [53]. Considering these factors (the frequent reports of antibiotic shortages, the increasing number of alerts from regulatory authorities, and the critical clinical significance of these drugs), we selected the ATC group J01 as the primary focus of our analysis, evaluating patterns of supply disruptions in several states from three distinct geopolitical regions (the EU, USA, and the Middle East).

**Figure 4:**
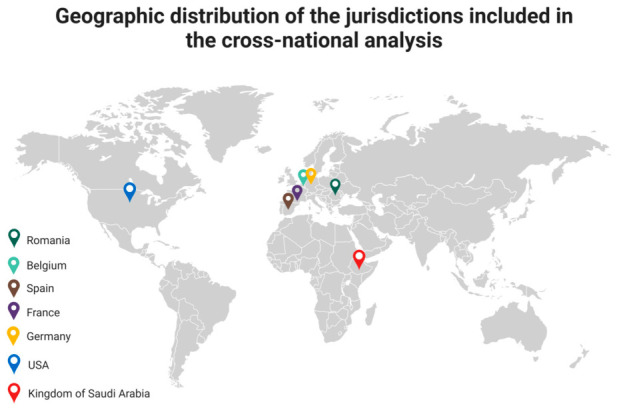
Geographic distribution of the seven jurisdictions included in the cross-national analysis of systemic antibiotic shortages. The study included national shortage registries from Belgium, France, Germany, Romania, Spain, the United States, and the Kingdom of Saudi Arabia.

Data on supply disruptions were sourced from publicly available national shortage registers. These registers were accessed through the EMA portal, which provides links to the national registers of the competent authorities in the Member States of the EU, as well as to other relevant databases on drug shortages. Seven jurisdictions were included in this analysis: Belgium [56], France [57], Germany [58], Romania [59], Spain [60], the United States (FDA drug shortage database) [61], and the Kingdom of Saudi Arabia [62]. These jurisdictions were selected to ensure geographic and regulatory diversity while maintaining access to publicly available and relatively well-structured national shortage registries, thereby improving the comparability of shortage data across different pharmaceutical markets and healthcare systems. The inclusion of both Western and Central–Eastern European countries enabled the exploration of regional variability in shortage reporting patterns, while the inclusion of the United States and the Kingdom of Saudi Arabia extended the analysis beyond the European context and increased the external relevance of the findings. This approach also facilitated the identification of potential structural vulnerabilities affecting the global antibiotic supply chain. The geographical distribution of the included jurisdictions is presented in Figure 4. Records corresponding to antibiotics classified under the ATC group J01 were subsequently extracted from each registry.

### 4.2. Data Harmonization and Construction of the Master Dataset

We manually reviewed, cleaned and harmonized the data extracted from national registries using Microsoft Excel. Differences among registry data in terms of active substance names, route of administration descriptions, and ATC classification levels were standardized using the names provided in the Anatomical Therapeutic Chemical classification system and EU terms.

We constructed a master dataset containing one record for each antibiotic, identified by its INN reported in at least one shortage registry. The initial data extraction yielded 350 product-level shortage records collected across the seven analyzed jurisdictions. These records were subsequently harmonized according to the WHO ATC/DDD classification system in order to identify unique antibiotic active substances (INNs). Following harmonization, 64 unique INNs were identified and used as the analytical unit for the statistical analyses evaluating multinational shortage recurrence patterns. Individual INNs could appear multiple times in the initial dataset due to reporting across different jurisdictions and commercial products. For each antibiotic, we recorded the countries where shortage was reported, and generated additional variables necessary for statistical analysis. Discrepancies in INN names across the analyzed registries were resolved by manually verifying each record that contained both the active ingredient name and the product’s trade name. INN standardization was performed using the World Health Organization’s Anatomical Therapeutic Chemical (ATC) classification (WHO ATC/DDD Index) [63]. For the records in which the ATC codes were not included in the national registries, they were added manually based on the official correspondence between the INN and the WHO ATC classification to ensure homogeneity in the therapeutic classification. Combination products (e.g., amoxicillin/clavulanate) were included in the analysis after harmonization according to the WHO INN and ATC/DDD nomenclature and were handled as distinct therapeutic entities. Combination antibiotics were not merged with single-agent formulations during the statistical analyses, and each combination was retained as a separate product category under its corresponding ATC classification code. The process of identifying duplicate records included manual verification of variables available in the registries, such as trade name, dosage form, strength, and package size. These characteristics allowed for differentiation between distinct products belonging to the same INN and facilitated the removal of any duplicates generated by multiple reports of the same product.

The different dosage forms, strengths, and commercial presentations of the same INN were kept as separate records during data collection, as each represented a distinct shortage event reported in the official registry. For the sake of statistical analysis, the unit of analysis was the INN (active ingredient). Consequently, variables such as the number of countries reporting a shortage and the multinational shortage indicator were derived and assigned at the INN level, ensuring that each active ingredient was counted only once in the analytical dataset. This approach was chosen because supply disruptions are ultimately determined by the availability of the active ingredient, rather than by a specific commercial formulation; consolidating data at the INN level, therefore, allows for a more accurate assessment of the true geographic extent of the shortage and avoids overrepresentation of molecules with a larger number of commercial products on the market. Furthermore, analysis at the INN level allows for meaningful comparisons across jurisdictions, given that the same active substance may be marketed under different trade names and in different formulations from one country to another. Data regarding the onset of shortage, its duration, or the estimated date of resumption of marketing were frequently missing or reported inconsistently across jurisdictions, which limited the ability to conduct additional longitudinal or survival analyses. Therefore, the analysis focused on variables comparable across registries, particularly the presence or absence of shortage for each INN and their distribution across jurisdictions. To be included in the analyses of this study, for each reported shortage, the availability of a minimum set of information necessary to validate each record was verified, namely INN, trade name, dosage form, and packaging characteristics. Data on shortages was collected exclusively from the official databases of regulatory authorities in the selected jurisdictions to ensure the methodological integrity and comparability of information across markets.

### 4.3. Variables and Derived Indicators

For the comparative analysis, several variables were defined in the main dataset, as follows: Country indicators: for each country included in the analysis, a binary variable was created indicating whether the antibiotic in question was reported to be in shortage in that jurisdiction; number of countries reporting shortage: for each antibiotic, the total number of countries in which shortage was reported was calculated by summing the indicators specific to each country. This variable reflects the degree of transnational recurrence of shortages: a multinational shortage indicator is a binary variable used to differentiate local shortages from those with multinational recurrence, which has a value of 1 if the antibiotic was reported in three or more countries or a value of 0 if the antibiotic was reported in one or two countries. The threshold of three jurisdictions was chosen to identify shortages that likely reflect vulnerabilities in international supply chains rather than isolated events at the national level. The core role of this indicator is to characterize shortage of structural relevance across jurisdictions. Another binary indicator (core shortage) was based on the geographic spread of each shortage signal at the INN level. Since the analysis included data from seven regulatory jurisdictions (Belgium, France, Germany, Romania, Spain, the U.S./FDA, and Saudi Arabia), supply disruptions reported in at least three jurisdictions (≥3 out of 7) were categorized as core shortages, reflecting situations affecting a significant portion of the markets surveyed. The choice of this threshold allowed for differentiation between isolated or locally specific shortage and those with cross-jurisdictional distribution, which may point to structural weaknesses in the supply chain, manufacturing capacity, or market accessibility. The use of a relative cutoff point, equivalent to a significant share of the included jurisdictions, ensures both a conceptual interpretability and an appropriate distribution of cases for further statistical analysis.

The EMA critical medicine status is a variable that indicates whether the antibiotic is included in the list of critical medicines published by the European Medicines Agency (EMA). This variable takes the value 1 if the antibiotic is included in the EMA list of critical medicines or the value 0 if it is not included in the list of medicines with critical status. This index was used to assess whether drugs considered essential for health systems are more likely to experience recurrent shortages. Furthermore, in order to assess the vulnerability of antibiotics predominantly used in hospitals, another binary variable was constructed, which serves to identify antibiotics included in the EMA list of critical medicines and administered intravenously. In this context, the variable takes the value 1 if the antibiotic analyzed is administered intravenously or 0 if it has another route of administration.

The route of administration was also classified in a binary variable, with a value of 1 for antibiotics administered by injection (intravenous or intramuscular) or 0 for other routes of administration. Each antibacterial agent was classified according to its therapeutic class (ATC level 3) to enable the assessment of shortage patterns among different antibacterial subclasses. During the data collection process, each shortage record was mapped to the specific product level using both the INN and the trade name reported in the official registries of regulatory authorities. During the analysis step, the primary unit of analysis was harmonized at the INN level to enable comparability across jurisdictions and to characterize the structural shortage patterns of J01-class antibiotics. Thus, the trade name was used to identify and validate each reported shortage record, avoiding duplicate records and enabling the consolidation of information at the active substance level. This method allows for the capture of shortage signals independently of the number of commercial products affected, reflecting disruptions in the market availability of the antibiotic without limiting the study to a specific manufacturer or brand.

### 4.4. Statistical Analysis

Statistical analyses were performed using JASP version 0.97 (JASP Team, Amsterdam, The Netherlands) [64] and Microsoft Excel version 2508 (Microsoft Corporation, Redmond, WA, USA) [65].

In the first stage, a descriptive analysis was conducted to characterize the distribution of shortages for antibacterial agents in the ATC group J01. The number of jurisdictions in which shortage was reported for each antibiotic was explored using descriptive statistics (mean, standard deviation, distribution) and graphical representations to illustrate the geographical reach of shortage.

To assess the association between critical drug status according to the European Medicines Agency (EMA) list and the multinational recurrence of shortage, contingency table analysis and the chi-square test (χ^2^) were used. The magnitude of the effect was estimated using Cramer’s V coefficient, interpreted according to conventional thresholds for effect size. Differences in the geographic extent of shortage between antibiotics classified as critical by the EMA and non-critical ones were assessed using the nonparametric Mann–Whitney U test, as the number of shortage countries for each INN did not follow a normal distribution. The effect size was estimated using the rank–serial correlation. The distribution of values was plotted using a raincloud plot to simultaneously illustrate the distribution, dispersion, and individual values.

To assess whether the proportion of EMA-critical antibiotics differs across the jurisdictions included in the analysis, the chi-square (χ^2^) test was used. The magnitude of the effect was estimated using Cramer’s V coefficient.

To identify factors independently associated with the occurrence of shortage with multinational distribution, a multivariate logistic regression model was constructed, using as the dependent variable the binary coding of reporting of supply disruption in at least three jurisdictions. The model included the following predictors: critical drug status according to the EMA; injectable route of administration. The independent variables were conceptualized as binary categorical variables. Because we did not have easy access to known drivers of shortages, such as the number of manufacturers or the age of the molecules (in each of the jurisdictions), this model is exploratory rather than causal.

Model performance was evaluated using fit indicators, including deviance, Akaike Information Criterion (AIC), Bayesian Information Criterion (BIC), McFadden R^2^, Nagelkerke R^2^, Cox and Snell R^2^, and Tjur R^2^. The model’s discriminatory power was assessed using the area under the ROC (Receiver Operating Characteristic) curve, expressed as the AUC (Area Under the Curve). For each predictor, the odds ratio (OR) and 95% confidence intervals were reported.

## 5. Limitations

The analysis was limited to assessing recurrence patterns between countries due to the heterogeneity of temporal information available in national shortage registers. The registers analyzed show significant differences in the availability of temporal indicators, such as the date of onset of the shortage, the classification of the shortage as permanent or temporary, and the date of resumption of availability for temporary shortages. In some registers, this information is only partially available, and in others it is completely missing. For this reason, it was not possible to perform analyses based on the duration of shortages or their temporal evolution. Therefore, the analysis focused on transnational recurrence patterns and the structural characteristics of antibiotics involved in shortages, rather than on the temporal dynamics of this phenomenon. For the same reason, another limitation of this study is related to the lack of standardized and comparable temporal indicators across the national registries analyzed. This lack of standardized tools made it impossible to systematically assess the impact of major events (e.g., COVID-19 pandemic) on the frequency or distribution of the antibiotic shortages in our dataset. At the same time, the lack of comparable temporal indicators leads to the identification of another limitation related to the heterogeneity of temporal reporting across national shortage registries. Because consistent date-level information was not uniformly available for all jurisdictions, the concept of “multinational recurrence” should be interpreted cautiously as the repeated identification of shortages across multiple territories rather than as evidence of temporally synchronized shortage events.

In the same line, variables such as manufacturer dependency, market concentration, or API supplier distribution were not publicly available in a standardized form across jurisdictions and, therefore, could not be included in the present model. Future studies integrating such variables may provide additional insights into the structural determinants of antibiotic shortages.

Another limitation is related to the fact that the medicines included on the EMA’s list of critical medicines are largely used in clinical practice and are often subject to closer monitoring by regulatory authorities. This may influence the likelihood of reporting shortages in the national registries. Therefore, our results should be interpreted in terms of an association between critical drug status and the multinational recurrence of shortage, without claiming or implying a direct causal relationship between the two. The observed differences may reflect both the clinical importance of these antibiotics and the high level of attention paid to their availability by the health authorities.

Our study utilized publicly available data from official shortage-reporting registries, which limited the inclusion to countries with transparent and structured systems for monitoring shortages. Consequently, the analysis encompassed seven jurisdictions with relatively well-developed reporting mechanisms. While these countries provided a set of data useful in identifying comparative patterns, generalization to other regions should be made with caution, particularly to low- and middle-income countries (LMICs), where public shortage registries are often nonexistent or limited in scope, accessibility, and standardization. The conclusions of our study reflect primarily patterns observable in health systems with functional reporting mechanisms and should be interpreted within this context.

## 6. Conclusions

Our findings indicate that supply disruptions for systemic antibiotics are frequently reported in national shortage registers. While some of these reports are limited to a single jurisdiction or a small number of countries, others refer to multiple antibiotics experiencing multinational recurrence, suggesting the existence of common vulnerabilities in international supply chains. Our analysis shows that antibiotics included in the EMA list of critical medicines updated in 2025 are more frequently involved in shortages reported across multiple territories, highlighting their vulnerability within international supply chains. This underscores the critical importance of continuously monitoring the availability of essential antibiotics. It would be ideal to develop efficient mechanisms to prevent and manage supply disruptions for these medications, which have consistently demonstrated their significance in everyday clinical practice. The findings from this study enhance our understanding of the dynamics of shortage in systemic antibiotics and underscore the need to develop multidisciplinary and multi-country strategies to prevent drug shortages in the global pharmaceutical market.

## Figures and Tables

**Table 1:** Association between EMA critical antibiotics and multinational shortages ^1^.

Multinational Shortage		Ema Critical Status		Total
		No	Yes	
No	Count	66.00	42.00	108.0
	% within column	62.86%	17.14%	30.86%
Yes	Count	39.00	203.0	242.0
	% within column	37.14%	82.86%	69.14%
Total	Count	105.0	245.0	350.0
	% within column	100.00%	100.00%	100.00%

^1^ Multinational shortage is defined as antibiotics reported in shortages in ≥3 countries.

**Table 2:** Distribution of EMA critical antibiotics across national shortage registries.

EMA Critical Status		Country							Total
		Belgium	France	Germany	Romania	Saudi	Spain	USA/FDA	
No	Count	13.00	7.00	13.00	10.00	47.00	10.00	5.00	105.0
	% within column	29.55%	31.82%	31.71%	22.22%	32.87%	31.25%	21.74%	30.00%
Yes	Count	31.00	15.00	28.00	35.00	96.00	22.00	18.00	245.0
	% within column	70.45%	68.18%	68.29%	77.78%	67.13%	68.75%	78.26%	70.00%
Total	Count	44.00	22.00	41.00	45.00	143.0	32.00	23.00	350.0
	% within column	100.00%	100.00%	100.00%	100.00%	100.00%	100.00%	100.00%	100.00%

**Table 3:** Logistic regression model summary for predictors of multinational antibiotic shortages.

Model	Deviance	AIC	BIC	df	ΔΧ^2^	*p*	McFadden R^2^	Nagelkerke R^2^	Tjur R^2^	Cox & Snell R^2^
M_0_	432.6	434.567	438.425	349			0.000		0.000	
M_1_ *	362.1	368.123	379.696	347	70.44	5.551 × 10^−16^	0.163	0.257	0.204	0.182

* M_1_ includes EMA critical status and injectable route.

**Table 4:** Logistic regression coefficients for predictors of multinational antibiotic shortages.

Model		Odds Ratio	*p*	95% Confidence Interval (Odds Ratio Scale)	
				Lower Bound	Upper Bound
M_0_	(Intercept)	2.241	3.125 × 10^−12^	1.786	2.811
M_1_	(Intercept)	0.653	6.098 × 10^−2^	0.419	1.020
	EMA critical status (1)	8.294	1.425 × 10^−15^	4.934	13.941
	Injectable route (1)	0.779	3.410 × 10^−1^	0.465	1.303

## Data Availability

The primary data used within this paper have been made publicly available at: https://figshare.com/s/a0bca1308da751f465b6 (accessed on 20 April 2026).

## Abbreviations

The following abbreviations are used in this manuscript:

ATC	Anatomical Therapeutic Chemical (classification)
AUC	Area Under the Curve
AIC	Akaike Information Criterion
BIC	Bayesian Information Criterion
CI	Confidence Interval
EMA	European Medicines Agency
EU	European Union
FDA	Food and Drug Administration
INN	International Nonproprietary Name
LMICs	Low- and Middle-Income Countries
OR	Odds Ratio
ROC	Receiver Operating Characteristic
SD	Standard Deviation
VIF	Variance Inflation Factor
WHO	World Health Organization
